# The Research and Development Thinking on the Status of Artificial Intelligence in Traditional Chinese Medicine

**DOI:** 10.1155/2022/7644524

**Published:** 2022-05-02

**Authors:** Nan Li, Jiarui Yu, Xiaobo Mao, Yuping Zhao, Luqi Huang

**Affiliations:** ^1^School of Electrical Engineering, Zhengzhou University, Zhengzhou 450001, China; ^2^China Academy of Chinese Medical Sciences, Beijng 100020, China

## Abstract

With the rapid development and application of artificial intelligence (AI) in medical field, the diagnostic ways of human health and the social medical structures have changed. Based on the concept of holism and the theory of syndrome differentiation and treatment, TCM realizes comprehensive informatization and intelligence with the help of AI technology in data mining, intelligent diagnosis and treatment, intelligent learning, and decision-making. Furthermore, the intelligent research of TCM technology will further promote the improvement in TCM diagnosis and treatment rules and the leaping development of TCM intelligent instruments. In this article, we performed a systematic review of scientific literature about TCM and AI. Moreover, the practical problems of TCM intellectualization, the current situation and demand of TCM, and the influence of AI in the TCM field are discussed by searching for literature using TCM scientific databases, reference lists, expert consultation, and targeted websites. Finally, we look forward to the application prospects of AI and propose a possible future direction of intelligent TCM in the current health-care system in China.

## 1. Brief Background

### 1.1. Traditional Chinese Medicine

Traditional Chinese medicine (TCM) is a complete medical knowledge system, which is based on the philosophy of Yin-Yang and Five Phases and essential Qi. It has accumulated rich theoretical and practical experience for thousands of years after long-term clinical practice and test [1–3]. TCM regards “inspection, auscultation, inquiring, and palpation” as the basic means for doctors to diagnose diseases and take Zang-Fu organs, meridian, blood, fluids, essence, and spirit as the basis of physiology and pathology [4, 5]. According to TCM theory, its connotation includes the theory of essential Qi, the combination of four diagnoses, the phenomena of body fluid and viscera, and the eight principles' pattern differentiation [6, 7]. The theoretical system of diagnosis and treatment with “syndrome differentiation and treatment” as the core and a large number of empirical data have become important resources for the research and development of TCM. The thoughts of integration and dialectical are the fundamental of TCM, which fully embodies the uniqueness of the TCM theory and the effectiveness of practice [8, 9]. It is the characteristics and advantages of TCM that is different from modern medical diagnosis and treatment system. Based on the concept of holism, TCM plays a unique role in advancing the development of life science and medicine.

On the other hand, with the dramatic increase in the prevalence of chronic conditions, the chemical medicines cannot fully meet the needs of health preservation, disease prevention, and treatment. Chemical medicines usually refer to the active ingredients extracted from natural minerals, animals, and plants as well as drugs prepared through chemical synthesis or biosynthesis. The experiences and knowledge in TCM can play a great role in human health and the large-scale development and application of natural medicines. TCM not only contains herbal plants, but also includes Chinese herbal extracts and minerals. Chinese herbal medicine is considered as an important supplement and substitute medicine in the Western countries as they are generally extracted from natural products with mild therapeutic effects, few side effects, and no artificial additives [10, 11]. Artemisinin is a sesquiterpene lactone drug having a peroxy group extracted from *Artemisia annua*, so it can be considered a chemical medicine. Tu Youyou, a researcher in TCM, has successfully extracted artemisinin and saved millions of lives around the world and won the 2011 Lasker Award in clinical medicine and the 2015 Nobel Prize in Physiology or Medicine. In general, TCM is a medical system whose main content is to study the treatment of diseases and adapt to nature [12–14]. In recent years, considerable research efforts have been put into the attempt to objectify and quantify the TCM by employing modern intelligent technologies and data cloud classification approaches.

### 1.2. Current TCM System in China

Western medicine was mainly introduced into China in the 19th century and gradually dominated the Chinese market. At present, TCM and Western medicine are two major schools of medicine in China. The Chinese have realized that TCM and Western medicine have different characteristics and functions, which manage to combine the best practices of TCM with Western medicine [15, 16]. Generally speaking, the characteristics and functions of TCM and Western medicine are reflected in four aspects. TCM usually uses inductive synthesis method, while the Western medicine uses simplified analysis method. TCM believes that the human body is an organic whole and pays attention to individual differences, while the Western medicine pays attention to physiological or medical test indicators. Due to the differences in theoretical basis, TCM tends to observe human pathogenic laws from the aspects of whole, dynamics, relationship, time, nature, and ecology, while the Western medicine tends to observe the laws of human disease from the aspects of local, detail, individual, structure, and material. From the perspective of diagnostic means, TCM takes inspection, listening, smelling, inquiry, and palpation as its basic skills. Although the inspection of Western medicine also includes questioning, listening, and smelling, the most important method is mainly based on medical records, physical examination, and laboratory test results to diagnose disease by modern scientific instruments such as blood analyzer, ECG monitor, etc. TCM focuses on the research objects with the method of system synthesis, while the Western medicine studies the objects with the method of analysis and reduction. Furthermore, TCM takes the overall concept and syndrome differentiation and treatment as the basic point. On the contrary, the purpose of the Western medicine is to clarify the focus of local diseases. In terms of medicine drugs, TCM drugs are natural animals, plants, and mineral medicine, and the Western medicine drugs are mainly obtained by chemical extraction or biosynthesis. To truly integrate TCM and Western medicine, we must first have a correct understanding of TCM and Western medicine and realize that TCM is as scientific as Western medicine. The Chinese and Western medicine are medical science with two different theoretical systems formed under different historical and cultural backgrounds. Only the complementary advantages of the two kinds of medicine can form a unique theoretical system of integrated Chinese and Western medicine. It should be made clear that induction, deduction, analysis, and synthesis are not mutually exclusive and incompatible. Importantly, the TCM and Western medicine should keep pace with the times and constantly move toward modernization.

On the other hand, the Chinese medicine of minorities, such as Miao medicine, Hui medicine, and Tibetan medicine, are also formally practiced in the Chinese health-care system [17–19]. The China's medical security system based on a three-level system is established in the 1950s, including hospitals, health centers, and clinics. According to the analysis of national health statistics, TCM has used about 5% of medical institutions and personnel to complete about 12% of diagnosis and treatment services in China. The cost of TCM diagnosis and treatment is 11.5% lower than that of public hospitals and the per capita cost of hospitalization is reduced by 24%. Therefore, the precise and flexible diagnosis and treatment methods of TCM have made outstanding contributions to alleviating the “difficult and expensive medical treatment.”

### 1.3. Artificial Intelligence

Artificial intelligence is a comprehensive interdisciplinary subject based on the computer science, cybernetics, information theory, neuropsychology, philosophy, etc., which is a new emerging and developing frontier subject with new ideas, concepts, theories, and technologies [20, 21]. Nowadays, the main research fields of AI include expert system, machine learning, pattern recognition, automatic programming, intelligent decision system, artificial neural network, etc. Particularly, AI based on machine learning and deep learning ushered in a new upsurge [22].

As an important branch of computer science, AI has laid an important theoretical foundation and made great progress after more than 60 years of development. Nowadays, AI is widely used in the diagnosis of common clinical diseases in modern medicine. For example, Hirasawa et al. designed a CNN system capable of automatically identifying gastric cancer from a large number of endoscopic images, which is promising in aiding the diagnosis of gastric cancer [23]. Wang et al. summarized the research progress and clinical application value of AI in the investigation, early diagnosis, treatment, and prognosis of colorectal cancer, to provide a comprehensive theoretical basis for AI as a promising diagnostic and treatment tool [24].

The combination of TCM and AI began in the 1970s, with the birth of the first international expert system of TCM, which was named “TCM Guan Youbo Hepatitis Diagnostic and Treatment Procedures,” as a landmark event. In the decades since, TCM expert system has made progress with the help of clinical practice guidelines, database, and the knowledge of specialists, assisting in standardizing TCM diagnostic models. For example, Weng et al. explored the quantitative diagnosis of the blood stasis syndrome, wherein the changes in tongue bodies of 352 patients of the blood stasis syndrome and 218 patients of non-blood stasis syndromes were observed by the expert system of tongue inspection of TCM [25]. Wang et al. constructed a novel self-learning expert system for diagnosis in TCM by incorporating several data mining techniques, and five representative cases were diagnosed to evaluate the performance of the system [26]. Silva et al. described an expert system for supporting the TCM consultation process both in terms of gathering and managing the patients' personal and symptomatic data, and of obtaining accurate diagnoses and treatments under regulated and reviewed protocols [27]. Nowadays, TCM began to combine with neural network [28], database, and multimedia technology [29], and the enrichment of AI algorithms brought new opportunities for the development of TCM. For instance, Zhang et al. proposed an AI-based TCM auxiliary diagnosis system, which can diagnose 187 common TCM diseases and related syndromes [30]. Huan et al. proposed a body constitution recognition algorithm based on deep convolutional neural network, which can classify individual constitution types according to face images [31].

## 2. Current Situation and Demands

China's population aging has the characteristics of large population, fast aging speed, and long duration of aging peak. In 2019, China's 60-year-old population has exceeded 248 million, accounting for 17.8% of the total population [32, 33]. The demographers predict that the proportion of people over 60 will increase from 12% to 40% within 50 years (from 2000 to 2050) [34]. Furthermore, the number of elderly people over 65 will reach 400 million in China, accounting for 26.9% of the total population [35]. Therefore, China will have a population with the highest percentage of the elderly people in the world. This will inevitably bring a host of problems and challenges in medical and health aspects.

**Figure 1:**
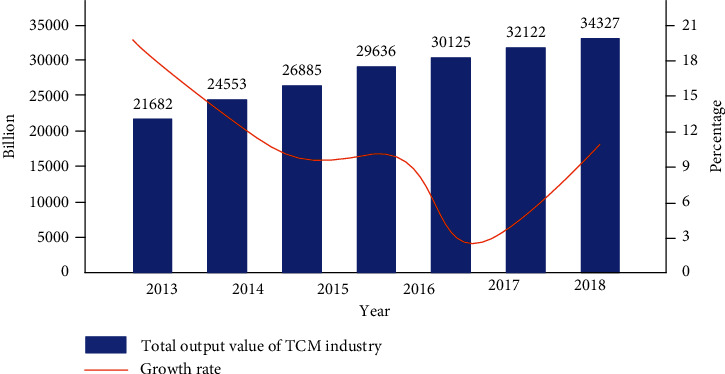
Total production value of TCM industry in 2013–2019.

Recently, the Chinese government has undertaken enormous efforts to modernize TCM by investing capital in scientific research and the economic value exploitation of TCM. The report of the 19th Session of National Congress of the Communist Party of China, held on October 18, 2017, once again put forward that “We will support both traditional Chinese medicine and Western medicine, and ensure the preservation and development of traditional Chinese medicine,” and upgrade to the level of “healthy China initiative,” which means that TCM has entered a new era from supporting development to inheriting development. The law of the People's Republic of China on TCM clearly points out that encourages scientific research institutions, colleges and universities, medical institutions, and pharmaceutical manufacturers to use modern science and technology and TCM methods to carry out scientific research on Chinese medicine, strengthen the integration exploration of Chinese and Western medicine, and promote the inheritance and innovation of TCM theories and technical methods. Meanwhile, the State Council of China issued the current situation and the strategic plan of the TCM development in 2016–2030. According to the survey, the establishment of intelligent TCM is mainly based on the following reasons:

1. Realize the “improvement in the service ability of community-level Chinese medicine” proposed in the 13th Five-year plan of TCM. According to the plan, more than 85% of the community health service centers and 70% of the township health centers will set up comprehensive service areas of TCM. The informatization need to be strengthened and the proportion of TCM diagnosis and treatment volume will strive to reach about 30% in the total medicine diagnosis and treatment. All community health service institutions, township hospitals, and 70% of village clinics will have the capacity of TCM service by 2021.
2. Accelerate the cultivation of TCM talents. At present, the doctors graduated from medical colleges are more inclined to identify diseases from Western medicine perspective in the process of disease diagnosis and treatment. In addition, the general practitioners in primary health institutions are most from Western medicine. Even many doctors who graduated from TCM colleges also have embarked on the road of westernization of TCM. In short, the Westernization of TCM is to replace the thinking of TCM with the thinking of Western medicine and then guide TCM to prescribe for patients. Therefore, the whole primary medical environment usually lacks a real atmosphere of TCM diagnosis and treatment. With the help of TCM intelligent system, it can shorten the training cycle of TCM talents, improve the supply speed of TCM professionals, and reduce the cost of training young doctors who graduated from a college of TCM. They usually focus on classroom learning, theoretical learning of TCM and Western medicine, and adopt the methods of learning from teachers, and oral and personal teaching. Students can follow teachers for consultation, prescription, and ward rounds. With the combination of theory and practice, teachers can demonstrate and guide students at any time, which help students better establish diagnosis and treatment thinking and use actual cases to understand the obscure theoretical knowledge in books.
3. Improve the service level of TCM hospitals' diagnosis and treatment. With the help of TCM intelligent medical association, the diagnosis and treatment level of medicine students, general practitioners, and TCM students will be greatly improved. The improvement in TCM diagnosis level not only meet the people's medical needs, but also enhance the confidence in TCM diagnosis and treatment. In particular, it is necessary to accelerate the construction and development of TCM, so TCM can better serve the health of the community-level people in China.
4. Expand the coverage of TCM services. The intelligent system of TCM has broad prospects and huge demands in clinical assistant diagnosis, telemedicine, and personal health management. The establishment of regional TCM intelligent medicine association cloud platform will help students to realize intelligent prescription and directly transmit diagnostic information to users as well as expand the coverage of TCM services. This online intelligent diagnosis modes of TCM are of great significance to the people who live in remote areas. Obviously, there is a broad prospect in theory and practice, which the demand of TCM intelligent system is getting more and more attention in China. The total production value of TCM industry in recent years is shown in Figure 1.

## 3. The Influence of AI in TCM Fields

AI is often used in expert medical system that is mainly a computer program designed by using the principle and method of expert clinical diagnosis to simulate the diagnosis process of TCM practitioners. It can help doctors to solve many complex medical problems, which effectively avoids misdiagnosis caused by human factors and improves the speed and accuracy of TCM diagnosis. For example, TCM intelligent robots can imitate human finger massage, precisely measure the hardness, achieve precise sensing and control of flexible soft tissues, and then calculate the pressure required for the massage by AI algorithm. They generate visual reports that allows doctors to use empirical data to measure the rehabilitation of patients and avoid misdiagnosis caused by human factors. In the field of TCM materials, the identification accuracy of TCM is improved by AI technology, near-infrared spectroscopy analysis, deep learning model, and multimodal fusion technology. Compared with traditional manual identification, multimodal fusion identification of TCM has shorter cycle and lower cost by image technology.

**Figure 2:**
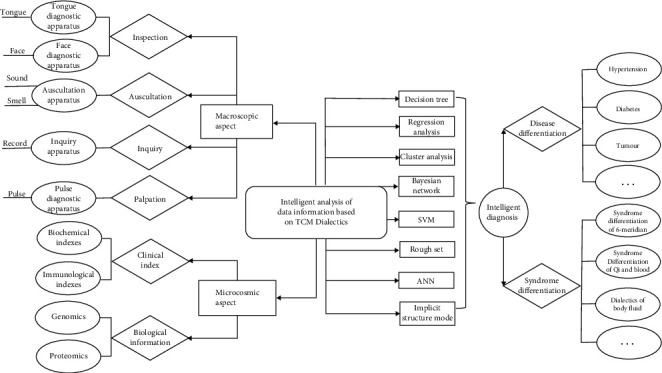
Intelligent analysis process of TCM.

On the other hand, intelligent TCM inherits and develops the medical theory and enrich clinical experience, which make the intelligent TCM diagnosis system very close to the diagnosis and treatment level of TCM experts. Furthermore, the process of intelligent diagnosis for the disease with single condition greatly promotes the inheritance of the experience of the famous Chinese medicine practitioner. The famous Chinese medicine practitioner refers to the outstanding representatives of the academic development of contemporary TCM. Their academic thoughts and clinical experience are the concentrated embodiment of the academic characteristics and theoretical characteristics of TCM. So, the application of AI technology is an intelligent treatment method integrating treatment and evaluation. The main achievements of the combination of TCM and AI can be divided into four categories: multisource data integration and construction, medical model change, experience inheritance, and intelligent instruments. The intelligent analysis process of TCM is shown in Figure 2.

### 3.1. Multisource Data Integration and Construction

TCM syndrome differentiation is a comprehensive description of patients' pathological state. The syndrome covers a large amount of dynamic information about human body, such as physical signs, tongue pulse, age, and constitution. It has multisource, multilevel, nonlinear, and strong uncertainty characteristics. Moreover, the big data of TCM also include the monograph of ancient Chinese medicine, literature journal, medical record monograph of famous and old Chinese medicine, medical record, community health file, wearable equipment data, astronomical and geographical data, etc. These data are scattered in different spatial locations, so it is difficult to collect and integrate them. The long and tedious process must be gradually integrated and accumulated. However, AI technology, such as text mining, web crawler, intelligent matching, etc., can quickly collect valuable knowledge in the text file and timely find the disease consistent with the symptoms of TCM. According to the characteristics of knowledge, different recording methods were taken. People will classify and manage the existing prescriptions, ancient books, and encoded TCM knowledge; comprehensively collect; and centrally edit. For some who are difficult to express, such as oral expression and personalized knowledge, we usually retain and input their original treatment process and retain some details in the treatment to the greatest extent. TCM doctors used AI models to establish standardized and unified diagnosis and treatment terms and realize language conversion. These intelligent methods are often suitable for converting symptom combination to diagnosis and treatment plan combination, so as to complete the function relation construction of *Y*_*i*_=*f*(*X*_*i*_).

These lines of information of different groups people can be collected, stored, and analyzed, then relevant diagnosis and treatment reports are given, forming a huge human symptom database. It can not only enrich and improve the exist clinical database, but also better promote the development of TCM digitalization. Large database is of great significance for the accuracy and sensitivity of TCM intelligent diagnosis. To some extent, TCM researchers can provide explanations for the scientific nature of TCM disease diagnosis and treatment by mining its potential objective laws from a large number of clinical data. The independent recognition, independent learning, independent judgment, and intelligent clinic treatment and diagnosis will be ultimately achieved with AI technology, database, and advanced algorithms. In addition, the construction of the database requires the collection and input of data as well as the characteristics of TCM.

The research of TCM should aim at the key points of scientific and technological innovation, such as biomedicine and system science, clarify the scientific connotation of TCM theory, and innovate and develop the theory and technology of TCM. New technologies such as information, biology, new materials, and emerging new methods are organically combined to strengthen the circular demonstration of the scientific nature of TCM data. The database construction should also take root in the clinical front line, strengthen the scientific nature of data, and explore the construction of evidence-based medicine system with the characteristics of TCM.

### 3.2. The Medical Model Change

With the maturity of information technology, the online and offline data fusion is effectively realized, which changes the traditional diagnosis and treatment mode of TCM. AI integrates a multidisciplinary integrated medical model and multipath diagnosis and treatment and makes up for the deficiencies of TCM data, information, technology so that personalized, fragmented, and fuzzy TCM clinical experience and knowledge have a more scientific expression. At present, the development of the field of “TCM + AI” mainly has several models: Chinese herbal medicine e-commerce, online consultation, O_2_O medicine delivery, health management, Chinese medicine media, Chinese medicine education, intelligent equipment etc. Specifically, the intelligent diagnosis equipments of TCM mainly use AI algorithms to simulate the diagnosis and treatment thinking of TCM to make a diagnosis and give a corresponding diagnosis and treatment plan. The different prescriptions can be provided for different diseases according to the analysis results of literature data, severity degree of disease, and patient situation. The doctor's task is to make choices and judgments based on their own clinical experience. For instance, Zhang et al. extracted the diabetes feature parameters from standard tongue image and established diagnosis model based on support vector machine algorithm [36]. Hu et al. studied the periodic characteristics of pulse waves in different blood pressure levels of the elderly people. The pulse data were preprocessed and pulse periods were segmented as well as the pulse wave features were evaluated by using feature selection classifier. The experience show that the selected features can be used for cardiovascular risk assessment based on pulse diagnosis of TCM [37]. Allwood et al. described the progress of AI in signal recognition and processing of hyperactive bowel sounds by extracting parameters from patients' cough and breath sounds [38]. The facts of TCM combined with AI indicate that the medical model can get better classification accuracy and provide a new thinking for diagnosis of various diseases. The intelligent diagnosis technology provides technical mean to evaluate the TCM therapeutic effects. The diagnosis and treatment can realize the objectification of facial color, tongue body, tongue coating, voice, pulse, and other symptom information. The standardization and quantification of TCM subjective symptom inquiry have a great significance to build database of TCM, which promotes the transformation of TCM tradition treatment mode to intelligence and information mode.

### 3.3. Experience Inheritance

AI technology can discover the correlation between the data in the massive TCM data and conduct training, learning, and optimization. Moreover, AI converts TCM classics and clinical diagnosis and treatment experience into data and form a large number of TCM databases. With the popularity of the Internet, the accumulation of data has accelerated. It provides a foundation for the application of industrial intelligence technology in the field of TCM. We should mine information from past data, especially the theories and clinical experience of well-known veteran TCM experts, and use scientific research methods to upgrade traditional experience to scientific law, so as to provide basis and ideas for further basic research. At the same time, the government has strengthened the supervision of data quality of TCM, improved the evaluation system, established internal and external linkage database, and comprehensively evaluated all kinds of data. In this way, the personalized and fragmented clinical experience of TCM has a scientific expression, and personal experience can be transmitted and exchanged. The model is adopted to determine the clinical diagnosis elements of prominent TCM experts, the structured data were transformed, and the diagnosis and treatment information database of TCM was summarized and established. For example, Cai et al. established TCMIO, a comprehensive database of TCM on Immuno-Oncology, which can be used to explore the molecular mechanisms of TCM in modulating the cancer immune microenvironment. All of these data, along with cheminformatics and bioinformatics tools, were integrated into the publicly accessible database [39]. Zhang et al. proposed the TCM-Mesh system, which records TCM-related information collected from various resources and could serve for network pharmacology analysis for TCM preparations. The database contains 6,235 herbs, 383,840 compounds, 14,298 genes, 6,204 diseases, 144,723 gene-disease associations, 3,440,231 pairs of gene interactions, 163,221 side effect records, and 71 toxic records, and web-based software construct a network between herbs and treated diseases, which will help to understand the underlying mechanisms for TCM [40]. Meanwhile, various auxiliary diagnosis and treatment systems and intelligent learning systems can improve talent training, TCM inheritance efficiency, and TCM diagnosis and treatment efficiency; integrate intelligence TCM into daily life; and truly achieve intelligent TCM.

### 3.4. The Development of Intelligent Instruments

The intelligent instruments are necessary to realize accuracy and rapid diagnosis of disease in the AI era. TCM attaches great importance to individual differences and needs to give syndrome differentiation diagnosis opinions according to the constitution of human body and the drug efficacy, but the diagnosis and treatment modes are easily affected by individual subjectivity or external factors. Compared with TCM diagnosis and treatment, precision medicine has seemingly different aspects. Syndrome differentiation and treatment is the essence of TCM diagnosis and treatment and the basic principle of TCM in understanding and treating diseases. Precision medicine includes precise detection, precise conditioning, precise diagnosis, and precise treatment. Under the guidance of the holism, it studies the precise correspondence and laws of human symptoms and signs, diseases, symptoms, etc. Therefore, the diagnosis and treatment concept of syndrome differentiation and precision medicine have similar starting point and focus. It also reflects the great connotation and breadth and depth of understanding of TCM in the process of disease diagnosis and treatment. The application of AI equipment can well solve this problem in TCM diagnosis aspects. The possible medical accidents caused by external factors can be avoided by objective and rigorous data processing.

**Figure 3:**
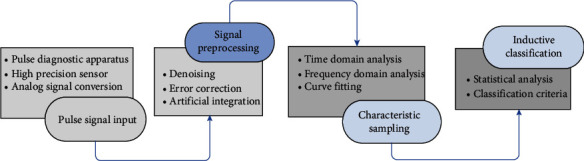
Pulse wave examination chart.

**Figure 4:**
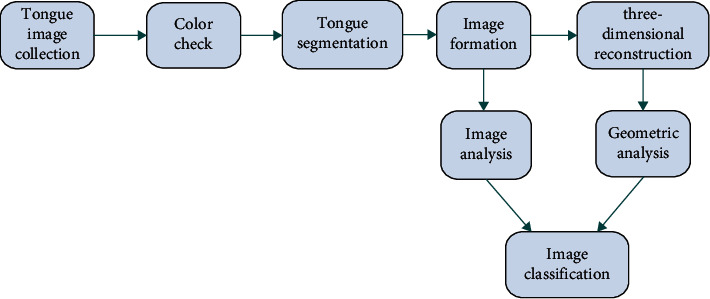
Tongue examination chart.

Pulse diagnosis and tongue diagnosis, as the basic links in the diagnosis of TCM, have attracted more and more attention. Pulse instruments mainly collect pulse information of patients through high precision sensors and conduct independent analysis and diagnosis according to unified standards. For example, professor Cui Ji has developed the pulse map identification standards of flat, smooth, and chord pulse by measuring the index range of pulse map characteristic parameters [41]. Zhang et al. proposed a graph-based multichannel feature fusion (GBMFF) method using multichannel features of wrist pulse information effectively [42]. On the other hand, tongue diagnosis also is the core component of TCM. The appearance of the human tongue conveys abundant valuable information for medical analysis. Abnormalities on tongue color and textures are commonly examined by medical professionals for either health status check or disease diagnosis. Tongue fissure, as a typical kind of texture anomaly, has been found closely associated with Melkersson-Rosenthal syndrome. Professor Zhang has developed a novel imaging system which records human tongue information faithfully and precisely for medical analysis [43]. The tongue body and tongue coating are separated, then tongue image features are identified and classified by AI technology. Wang et al. proposed an AI framework using deep convolutional neural network (CNN) for the recognition of tooth-marked tongue [44]. The specific process of pulse and tongue diagnosis are shown in Figure 3 and Figure 4, respectively.

## 4. The Prospect of Intelligent TCM

TCM workers are responsible for disseminating TCM knowledge and promoting Chinese culture. TCM hospitals account for the vast majority of TCM medical institutions and play a key role in providing TCM medical services and promoting TCM culture as well as technology. AI technology can detect the characteristics of most diseases by the massive TCM database, which make the ancient and modern TCM data be an endless precious resource rather than an empirical “garbage data” that are difficult to deal with. At present, the multitype database of TCM has become a solid information for TCM with its abundant data. The TCM stroke prevention database contains data on the treatment of stroke with TCM, acupuncture, and massage from 1996 to 2006. It has established epidemiological information, diseases, syndromes, and other related content and realized the analysis of four categories of factors including clinical research, diagnosis, treatment, and disease relationship as well as the purpose of disease-related data sharing services and data mining. Ai et al. screened the literature on TCM treatment of diabetic retinopathy through the Web of Science, ScienceDirect, PubMed, Google scholar, and CNKI online databases. In addition, they summarized and analyzed the prescriptions, herbs, and identified compounds of TCM on the treatment of diabetic retinopathy from the aspects of anti-inflammation, antioxidative stress, antiangiogenesis, and antiapoptosis [45].

With the improvement in TCM informatization and the development of AI technology, intelligent TCM is imperative for the construction of modern medical system with Chinese characteristics. The development of intelligent TCM system not only covers information release, medical service, health management, medical organization cooperation, and doctor-patient communication, but also innovates the medical service mode, builds the information sharing service system, and makes the medical service move toward the real sense of intelligence and convenience. Importantly, the advance of intelligent TCM should be organically combined with the construction of grassroots TCM and the development of smart city, such as building a “healthy TCM hut” model that promotes the improvement in grassroots TCM service level, which will bring new development opportunities for the overall industry of TCM.

## 5. Discussion and Conclusions

AI is the representative of the latest round of technological and industrial revolution. At present, it has developed to the fifth generation, which can handle massive information, carry out cloud computing and reasoning, and simulate human thinking with logic reasoning function. AI technology is mainly used in research and development of intelligent syndrome differentiation system and intelligent diagnosis and treatment equipment in TCM. The utilization of intelligent instruments and the medical records based on patients' condition can be used to determine the syndrome type of the disease and then select the responding treatment method. It expands the research scope of TCM and increases the research means, as well as enriches the methods of TCM.

On the other hand, the thinking ways and scientific theory of TCM are similar to the complex scientific thinking mode. Therefore, it is the classic representative of complex science, which is in accordance with the development direction of future science in some extent. AI has brought many opportunities for China's economic and social development, as well as great chances and challenges for TCM in future. We can learn and improve diagnostic means with the help of TCM big data, but also constantly inherit and innovate human experience. In terms of the development of intelligent TCM, I consider that it will probably go through three stages: digitalization, intellectualization, and popularization.

1. Ancient and modern TCM classics and experience are digitized, and AI is used in data mining and processing. All data are stored in the cloud system, including TCM classics, TCM clinical diagnosis and treatment data, human health status data, etc., which can be searched and obtained at any time and anywhere. With the help of TCM database, TCM doctors can quickly grasp the existing TCM experience and realize intelligent retrieval and consultation of TCM diagnosis and treatment plans.
2. The scale of TCM databases has gradually increased, and the level of intelligence has become more in-depth and proficient. The wide application of intelligent sensor technology, such as wearable devices, mobile phones, and smart terminals, makes patients use intelligent diagnostic instruments to diagnose and analyze independently user's physical state. At this stage, all kinds of intelligent diagnostic instruments will be developed and used to help people solve many practical problems.
3. Intelligent TCM will be integrated into daily life and become a part of human life. The modernization intelligent TCM of multidisciplinary, multilevel, and multimethods is established based on the cross cooperation. The defects of TCM dialectics' subjectivity and fuzziness are well avoided by various intelligent treatment models and advanced algorithms. In the process of disease diagnosis and treatment, intelligent TCM will be recognized and accepted by doctors and patients and gradually popularized throughout the country.

Despite its limitations, all medicine is a form of local knowledge that is a relevant part of human medicine. The medical knowledge of different nationalities and countries are digitized, which can gather the vast ocean of medical big database. Furthermore, the medicine systems also need to integrate the medicine wisdom of different nationalities and countries. In the era of AI technology and advanced algorithms, TCM, as an ancient science and art, is brought to a greater height for its ultimate mission of benefiting human health. There is no doubt that TCM will not only provide health services for the Chinese nation, but also will make an important contribution to the health of people all over the world.
